# Supplementary material for: Phosphorylated Fish Gelatin and the Quality of Jelly Gels: Gelling and Microbiomics Analysis
Source: Foods. 2023 Oct 7;12(19):3682. doi: 10.3390/foods12193682 (PMC10572387; doi:10.3390/foods12193682)
Supplement: Supplementary file 1 [file foods-12-03682-s001.zip › foods-2632723-supplementary.pdf]

Table S1 Specific composition of the JGs

Sample name	Gelatin(w/v)	Other ingredients(w/v)
FG:κC (2:1), /PFG:κC (2:1)	0.67% FG<br>0.33% κC	Sodium citrate 0.1%<br>Green tea essence 0.1%<br>potassium sorbate 0.1%<br>maltitol 18%<br>skim milk powder 16%
FG:κC (1:1) /PFG:κC (1:1)	0.5% FG<br>0.5% κC	
FG:κC (1:2)/PFG:κC (1:2)	0.33% FG<br>0.67% κC	

Table S2 Sensory scoring criteria

Project	Grading	Score
Color（15 points）	Uniform milky white or slightly yellowish	10-15
	Lighter milky white or Light yellow color slightly darker	5-10
	The color is too dark and uneven	0-5
Taste（30 points）	Smooth and tender taste with milky flavor	20-30
	Smooth and tender taste with no or less milk flavor	10-20
	The taste is rough and have great toughness	0-10
Textural acceptance（30 points）	Gelatinous, The texture is more uniform and soft and delicate	20-30
	Medium	10-20
	Not gelatinous, uneven texture and too soft or too hard	0-10
Hardness（15 points）	Good	10-15
	Rough	5-10
	Slightly worse	0-5
Elasticity（10 points）	Good elasticity	7-10
	Slightly lack of flexibility	4-7
	General or poor elasticity	1-4

Table S3 Changes in the total number of colonies in JGs

Storage time/day	Sample name	Total number of colonies/CFU/g	Sample name	Total number of colonies/CFU/g
	FG:κC (2:1)	15.0	PFG:κC (2:1)	33.3
0	FG:κC (1:1)	20.0	PFG:κC (1:1)	33.3
	FG:κC (1:2)	23.3	PFG:κC (1:2)	40.0
7	FG:κC (1:2)	33.3	PFG:κC (1:2)	80.0
14	FG:κC (1:2)	70.0	PFG:κC (1:2)	133.3
